# Adam T, Aliferis C, editors. Personalized and Precision Medicine Informatics: A Workflow-Based View: Springer Nature Switzerland AG; 2020; print ISBN 978-3-030-18625-8; online ISBN 978-3-030-18626-5

**DOI:** 10.3325/cmj.2020.61.577

**Published:** 2020-12

**Authors:** Nina Lukač

**Affiliations:** *nina.lukac@gmail.com*

Field of Medicine: Personalized and precision medicine (PPM)

Audience: The book provides a detailed overview of PPM workflows, targeting primarily informaticists. Nevertheless, it can be also useful to any health care professionals, students, or scientists interested in PPM.

Purpose: As outlined by the editors in the introductory chapter, the book was written with the following three objectives in mind: 1) “to review the present state of the art in PPM and the associated PPM informatics, and especially to map out all major forms of PPM and systematize them for better understanding and communication”, 2) “to review trends and future developments in PPM and PPM informatics and outline concrete areas of high value research in PPM informatics,” and 3) “to provide a consolidated and up to date PPM survey that can assist with the training of PPM researchers and practitioners (including informatics and non-informatics students and professionals).”

Contents: The book contents are divided into five parts, including an introductory chapter, three main parts, titled “Classical PPM,” “Emerging PPM,” and “Integrative Informatics for PPM,” and a conclusion.

The introductory chapter familiarizes the reader with the field and definition of PPM, outlines the book's purpose, and lists its main objectives. This chapter also provides a short overview of the contents of the three main parts.

The second part of the book, titled “Classical PPM,” addresses the well established use of PPM and associated informatics, namely informatics methods and workflows used for clinical risk assessment, outcome prediction and subsequent decision making, principles of clinical genetics and genetic counseling, and fundamentals of pharmacogenomics workflows. The first chapter concerning risk assessment provides an overview of early PPM modalities and the basics of disease severity and risk modeling, such as prediction models, their evaluation, new risk factor selection, and methods that facilitate model interpretation. The second chapter describes the principles of guideline-driven PPM informatics in a case study where an informatics framework for electronic health records is developed for clinical decision making in patients with hypertension. The chapter focusing on the principles of genetic counseling provides a general workflow for clinical genetic assessment and explains the terms used in genetic testing. It lists the fields where PPM informatics use is currently well implemented, brings to attention expanding and emerging roles of genetic counselors, and touches on practice guidelines, educational standards, ethical and legal considerations. The field of pharmacogenomics is dealt with in two separate chapters, the first of which provides an introduction to the field and a detailed description of two types of pharmacogenomics workflows in a learning health care system. The other provides an industry perspective, showing how the Mayo Clinic-cofounded startup OneOme and its RightMed pharmacogenomic model-based personalized prescription solution tackle the need for PPM use in drug prescription.

The third part of the book, titled “Emerging PPM,” presents the experience of several different authors with emerging PPM workflows. The introductory chapter familiarizes the reader with the concepts and workflows for the development and validation of predictive models for prognosis and risk stratification using big data, and describes the algorithms used in predictive modeling. A separate chapter introduces the concept of molecular profile and describes a workflow for molecular profile-based PPM, including the main principles of informatics used in model selection, fitting, and error estimation. This is followed by a case study detailing the use of this workflow in bevacizumab trials in patients with ovarian cancer. A chapter also describes the architecture of the National Cancer Institute (NCI) transcriptional pharmacodynamics workbench software, a tool that enables biomedical scientists to analyze cancer molecular profiles. Another chapter addresses the application of platform-independent isoform-level gene expression-based classification system (PIGExClass) in two cancer types, and examine the possibilities for isoform-level cancer gene subtyping. A separate chapter focuses on different next-generation sequencing methods used for cancer profiling in precision oncology, and discusses bioinformatic analysis, the main large-scale studies performed in the field, and existing data-sharing networks. The next chapter deals with the need for harmonization of omic data across PPM databases to create larger cohorts for discovery validation, describing the strengths and challenges of such an approach. Besides the examples of workflows used in precision oncology, there is also a chapter presenting the National Institute for Mental Health’s Research Domain Criteria initiative as an example of PPM use in psychiatry. Two chapters discuss the implementation of PPM in clinical trials. One describes how it can be used to overcome some of the obstacles in phase-IV clinical trials by integration of electronic health records, and briefly discusses innovative trial approaches, such as pragmatic trials and drug repurposing. The other focuses on precision trials informatics, meaning the use of PPM in clinical trial design where group assignment is based on biomarker profile. It describes the NCI molecular profiling-based assignment of cancer therapy study, the first study using precision medicine in clinical trials, and the GeneMed precision trial management system design and workflow. This part's last chapter deals with large-scale PPM implementation by Gesinger health care system, a rural integrated health care delivery system in Pennsylvania.

The fourth part, titled “Integrative Informatics for PPM,” presents general workflows for the use of genomic data in clinical workflow, the architecture of PPM informatics supporting clinical decision making, and current and future possibilities for education in the PPM field. The first chapter extensively describes the integration of omic data into the clinical workflow by using a model for data translation from bench to bedside, highlighting cost considerations and ethical and legal issues. The next chapter depicts the general architecture of PPM informatics for clinical decision making and its components, providing examples of single-institution and large-scale implementation of pharmacogenomics. Another paradigm of informatics architecture for large-scale PPM implementation is given in a separate chapter describing Geisinger’s ProvenCare model used in cardiac surgery. The fourth part ends with a chapter about PPM informatics training, which lists the prerequisites, core competencies, and various forms and levels of PPM education.

The fifth part summarizes the current state of PPM; the challenges of its large-scale implementation; touches on economics, education, and ethical and legal issues; and discusses future PPM opportunities.

Highlights: The review of the currently used and emerging PPM workflows provides the reader with a comprehensive and detailed survey of PPM. Various authors describe PPM workflows from informatics methods to implementation by a practicing physician. Besides workflow presentation, several chapters include case study examples. Detailed workflow descriptions are often supplemented by graphical representations, an approach that enables readers to more easily follow the text. Even though the book is written primarily for readers with a background in informatics, it can also be useful to any health care worker or scientist interested in PPM.

Related Reading: Important related reading from the field of PPM is cited in the reference section of the Introduction chapter. Additionally, every chapter contains its own reference section.

